# Overexpression of Cholesteryl Ester Transfer Protein Increases Macrophage-Derived Foam Cell Accumulation in Atherosclerotic Lesions of Transgenic Rabbits

**DOI:** 10.1155/2017/3824276

**Published:** 2017-11-28

**Authors:** Shoucui Gao, Xiaojing Wang, Daxing Cheng, Jiayan Li, Lu Li, Linwu Ran, Sihai Zhao, Jianglin Fan, Enqi Liu

**Affiliations:** ^1^Research Institute of Atherosclerotic Disease, Xi'an Jiaotong University Cardiovascular Research Center, Xi'an, Shaanxi 710061, China; ^2^Laboratory Animal Center, Xi'an Jiaotong University Health Science Center, Xi'an, Shaanxi 710061, China; ^3^Laboratory Animal Center, Ningxia Medical University, Ningxia 750004, China; ^4^Department of Molecular Pathology, Interdisciplinary Graduate School of Medicine and Engineering, University of Yamanashi, Yamanashi 409-3898, Japan

## Abstract

High levels of plasma high-density lipoprotein-cholesterol (HDL-C) are inversely associated with the risk of atherosclerosis and other cardiovascular diseases; thus, pharmacological inhibition of cholesteryl ester transfer protein (CETP) is considered to be a therapeutic method of raising HDL-C levels. However, many CETP inhibitors have failed to achieve a clinical benefit despite raising HDL-C. In the study, we generated transgenic (Tg) rabbits that overexpressed the human CETP gene to examine the influence of CETP on the development of atherosclerosis. Both Tg rabbits and their non-Tg littermates were fed a high cholesterol diet for 16 weeks. Plasma lipids and body weight were measured every 4 weeks. Gross lesion areas of the aortic atherosclerosis along with lesional cellular components were quantitatively analyzed. Overexpression of human CETP did not significantly alter the gross atherosclerotic lesion area, but the number of macrophages in lesions was significantly increased. Overexpression of human CETP did not change the plasma levels of total cholesterol or low-density lipoprotein cholesterol but lowered plasma HDL-C and increased triglycerides. These data revealed that human CETP may play an important role in the development of atherosclerosis mainly by decreasing HDL-C levels and increasing the accumulation of macrophage-derived foam cells.

## 1. Introduction

Epidemiological studies have clearly shown that a low high-density lipoprotein cholesterol (HDL-C) level is a strong and independent risk factor for the development of cardiovascular disease (CAD) [1]. Cholesteryl ester transfer protein (CETP) transfers the cholesteryl esters from HDL to apolipoprotein B- (apoB-) containing particles in exchange for triglycerides (TG) [2] and has been considered to be a new drug target for increasing HDL-C levels. Pharmaceutical CETP inhibitors such as Torcetrapib [3] and Dalcetrapib [4] have been shown to raise HDL-C levels effectively, but research into their clinical efficacy was unfortunately terminated due to off-target effect or lack of clinical benefit. A meta-analysis suggested that Evacetrapib, either as a monotherapy or in combination with a statin, reduces low-density lipoprotein cholesterol (LDL-C) and increases HDL-C levels without affecting TG concentrations [5] but has no clinical benefit [6]. The newer CETP inhibitors, Anacetrapib and TA-8995, have shown promising effects on the lipid profile and metabolism (increase in HDL-C and reduction in LDL-C levels), but their cardiovascular effects and safety profile have not yet been confirmed in large outcome trials [7]. Despite an increase in HDL-C and a reduction in LDL-C, treatment with Torcetrapib and Dalcetrapib was aborted due to an increase in the risk of major cardiovascular events and mortality [8]. Studying common CETP gene variants has not yet led to a consensus on the connection between CETP and atherosclerosis, and the relationship between reduced CETP function and susceptibility to atherosclerosis has proven complex and confusing [9–13]. Most but not all studies in transgenic (Tg) mice have shown that CETP inhibition reduces atherosclerosis development [14–18], and the role of CETP in atherosclerosis requires further deep investigation because of the differences in the lipid metabolism of mice and humans. Rabbits have plasma LDLs and are more susceptible to atherosclerosis than rodents, which are relatively resistant to atherosclerosis [19]. Inhibition of CETP in cholesterol-fed rabbits led to increased HDL-C levels and reduced atherosclerotic lesions but had no effect on aortic cholesterol content [20–23]. However, whether the overexpression of CETP will affect plasma lipoproteins, atherosclerotic lesions and plaque composition in cholesterol-fed rabbits is unclear. In our study, we created Tg rabbits expressing human CETP (hCETP) transgene to investigate the effect of CETP on atherosclerotic lesions and lipoprotein metabolism. Our results showed that increased expression of hCETP increased the accumulation of macrophage-derived foam cells in atherosclerotic lesions.

## 2. Materials and Methods

### 2.1. Generation and Identification of Human CETP Transgenic Rabbits

**Figure 1:**
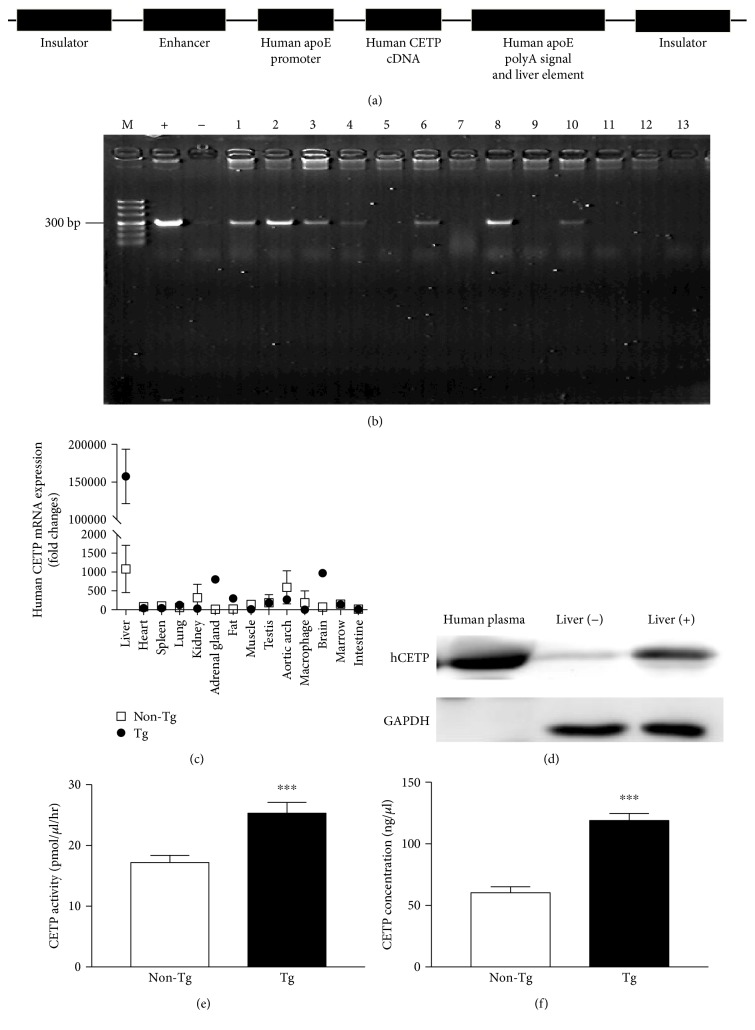
Generation and identification of human CETP Tg rabbits. (a) Tg construct for the microinjection. (b) Identification of the integration of the human CETP transgene in the rabbit genome by PCR (M: DNA marker; +: positive control plasmid; −: negative control; lanes 1–13: rabbit DNA sample). (c) Tissue distribution of human CETP mRNA in Tg and non-Tg rabbits (*n* = 3 for each group). (d) Western blotting analysis of CETP from liver and plasma (*n* = 3 for each group). (e) Plasma CETP activity (*n* = 4 for each group). (f) Plasma human CETP concentrations (*n* = 7 for each group). Data are expressed as the mean ± SEM. ^∗∗∗^*P* < 0.001 versus non-Tg littermates.

Japanese white rabbits were supplied by the Laboratory Animal Center of Xi'an Jiaotong University. The generation of Tg rabbits expressing human CETP (hCETP) was conducted in our laboratory by microinjection as previously described [24]. For hepatic expression of hCETP, a 1717 bp cDNA of the (NM_000078) hCETP gene was cloned into EcoRV and SacII sites 3′ of the human apoE promoter and 5′ of the human apoE poly A signal and liver element. The resultant fragment was isolated by digestion with Sal I (Figure 1(a)), injected into fertilized rabbit zygotes, and then transplanted into recipient rabbits. Through polymerase chain reaction (PCR) of the genomic DNA extracted from the blood, founder Tg was identified and then bred into F1 progeny. Four-month-old Tg and non-Tg rabbits were used for the current study. Both Tg rabbits (*n* = 12) and non-Tg rabbits (*n* = 12) were fed a chow diet containing 0.3% cholesterol and 3% soybean oil for 16 weeks. All animals were sacrificed by an overdose of pentobarbital sodium and xylazine hydrochloride. All animal experiments were approved by the Laboratory Animal Administration Committee of Xi'an Jiaotong University and performed according to the Guidelines for Animal Experimentation of Xi'an Jiaotong University and the Guide for the Care and Use of Laboratory Animals published by the US National Institutes of Health (NIH, Publication number 85–23, revised 2011).

### 2.2. Biochemical Analyses

Blood samples were collected via the auricular artery using an EDTA anticoagulant tube after overnight fasting and then centrifuged (3000 rpm, 15 min, 4°C) to obtain the plasma. The plasma TG, total cholesterol (TC), LDL-C, and HDL-C were analyzed every 4 weeks using commercial kits (Biosino Bio-Technology & Science Inc., Beijing, China). Plasma CETP activity was determined as previously described [25]. The plasma CETP concentrations were measured using a human cholesteryl ester transfer protein ELISA kit (Cusabio Co. Ltd., Hubei, China) according to the manufacturer's instructions.

### 2.3. Measurement of Blood Pressure

The blood pressure of the rabbits was measured as previously described [24]. First, rabbits were anesthetized with pentobarbital sodium. Then, an artery catheter was inserted into the ear artery with a pressure transducer and amplifier attached to a digital PowerLab data acquisition system (ML870 PowerLab) (AD Instruments, Bella Vista, NSW, AUS). The data were collected 10 minutes after the rabbits became calm and there were no blood pressure fluctuations. The blood pressure measurements were calculated using Chart 5 Pro v5.5 software (AD Instruments).

### 2.4. Quantitative PCR Analysis

Total RNA was isolated from the liver, heart, spleen, lung, kidney, adrenal gland, fat, muscle, testis, aortic arch, macrophage, brain, marrow, and intestine of rabbits using TRIzol reagent (Invitrogen, CA, USA) and reverse-transcribed into cDNA using a reverse transcription kit (Takara, Shiga, Japan). The quantitative real-time PCR reactions were composed of SYBR® Premix Ex Taq™ II (10 *μ*l), total primer pairs (2 *μ*l), cDNA template (1 *μ*l), and RNase-free water (7.0 *μ*l). The primers used for real-time PCR were as follows: human CETP primers: forward, 5′-TCAGCCACTTGTCCATCGC-3′; reverse, 5′-GGCATCGGTCCGCACTCTA-3′and rabbit GAPDH primers: forward, 5′-ATCACTGCCACCCAGAAGAC-3′; reverse: 5′-GTGAGTTTCCCGTTCAGCTC-3′. The cycling conditions were 95°C for 30 s, followed by 40 cycles of 95°C for 30 s, and 55°C for 40 s.

### 2.5. Western Blotting

Protein samples were extracted from the fresh livers of both Tg rabbits and non-Tg littermates (*n* = 3) incubated in a lysis buffer (20 mM Tris-HCl, 150 mM NaCl, 1 mM EDTA, 1 mM EGTA, 1% Triton X-100, and protease inhibitor, pH 7.4) for 30 minutes in ice and then centrifuged for 10 minutes at 12000g to discard the cell debris. Total protein concentrations were determined to ensure that the equal loading of proteins was separated on 10% SDS-PAGE and transferred onto PVDF membrane. Antibodies against rabbit CETP (1 : 400; Abcam, Cambridge, UK), human CETP (1 : 400; Abcam, Cambridge, UK), and GAPDH (1 : 500; Beyotime, Beijing, China) were used for Western blot analysis. The blots were developed using HRP-conjugated secondary antibodies (1 : 2000; Thermal, MA, USA) and the ECL-plus system.

### 2.6. Atherosclerosis Quantification

The entire “aortic tree” fixed in 10% neutral buffered formalin was stained with Sudan IV for evaluation of the gross atherosclerotic lesions as previously described [26]. The area of the atherosclerotic lesion (sudanophilic area) was measured using image analysis software (Mitani, Tokyo, Japan) [27]. For the microscopic quantification of the lesion area, the aortic arch was processed through routine steps of desiccation followed by clearing, dipping and embedding in wax, and serial sectioning (4 *μ*m). The sections were then stained with hematoxylin and eosin (H&E) and Elastica van Gieson (EVG). The lesion composition of the atherosclerosis plaque was evaluated after immunostaining with the anti-rabbit *α*-actin antibody (1 : 500; Dako, CA, USA) for the identification of smooth muscle cells and the anti-rabbit RAM11 antibody (1 : 100; Dako, CA, USA) for the identification of macrophages as previously described [28]. The sections for microscopic quantification were examined and photographed under a light microscope equipped with a digital camera (Nikon, Tokyo, Japan) and measured with image analysis software (WinROOF ver. 6.5, 130 Mitani. Fukui, Japan).

### 2.7. Statistical Analysis

In a total, 24 rabbits (*n* = 12 for each group) were used for the current study to examine the effect of increased plasma CETP on plasma lipids and atherosclerosis. For lipid analysis and atherosclerosis evaluation, all rabbits were used. For other analyses, only some of the rabbit specimens were collected and used: CETP levels (*n* = 7 for each group) by ELISA, CETP activity (*n* = 4 for each group), CETP mRNA, and protein expression by RT-PCR and Western blotting analysis (*n* = 3 for each group) were quantitated for a comparison. Data are expressed as the mean ± SEM. Statistical analysis was performed using Student's *t*-test with an equal *F* value or Welch's *t*-test when the *F* value was not equal. *P* < 0.05 was considered statistically significant.

## 3. Results

### 3.1. Identification of Human CETP Tg Rabbits

In this study, we successfully generated Tg rabbits (Tg) expressing hCETP confirmed by PCR genotyping (Figures 1(b) and 1(c)). Founder Tg rabbits were mated with non-Tg rabbits, and the germline transmission was confirmed. As shown in Figure 1(c), human CETP transgene was almost exclusively expressed in the liver whereas no expression in non-Tg rabbits. The plasma and hepatic CETP expression was evaluated by Western blotting analysis (Figure 1(d)) and Tg rabbits expressed two-fold higher levels of CETP concentrations and activity (Figures 1(e)–1(f)) than non-Tg rabbits.

### 3.2. Plasma Biochemical Parameters

**Figure 2:**
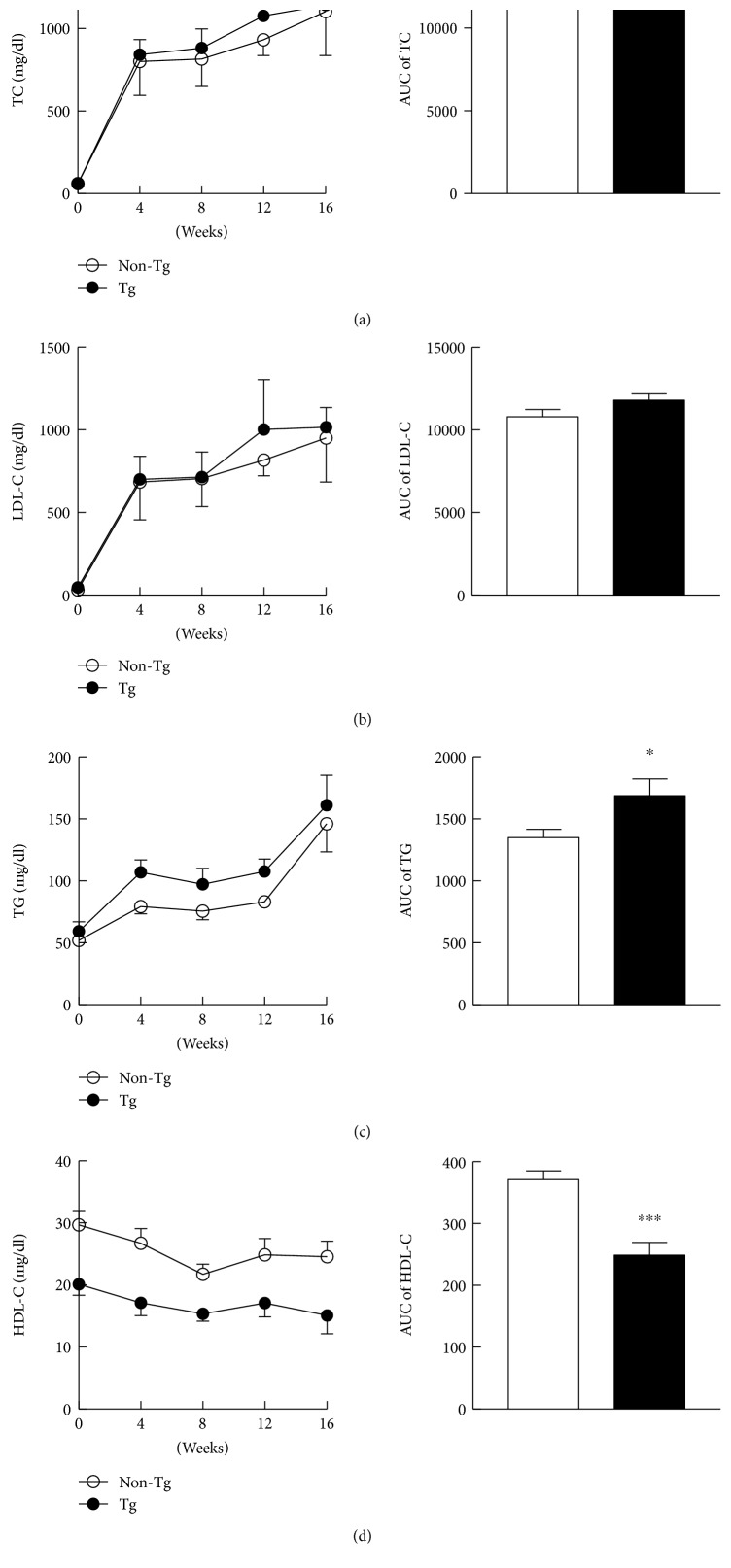
The plasma levels of total cholesterol (TC), low-density lipoprotein cholesterol (LDL-C), triglyceride (TG), and high-density lipoprotein cholesterol (HDL-C) (a–d). Data are expressed as the mean ± SEM, *n* = 12 for each group. ^∗^*P* < 0.05, ^∗∗∗^*P* < 0.001 versus non-Tg littermates.

Plasma levels of lipids were measured every four weeks as shown in Figure 2(a). For calculating lipid levels during the experiment, plasma lipids were also expressed by the area under the curve (AUC) shown in Figure 2(b). Both mean values and AUC of the plasma TC and LDL-C after high cholesterol diet (HCD) were not significantly different between two groups. Throughout the experiment, the TGs (Figure 2(c)) were maintained at higher levels in Tg group than in non-Tg group, while the HDL-C levels (Figure 2(d)) were significantly lower in Tg rabbits.

### 3.3. Body Weight, Organ Weight, and Blood Pressure

The effects of hCETP on the body weight, organ weight, and blood pressure are shown in Table 1. There was no obvious difference in the body weight between two groups, either at the start or at the end of the experiment. Neither the weight of the major organs, including the heart, kidneys, and liver, nor the blood pressure was significantly different between two groups.

### 3.4. Quantification of Atherosclerotic Lesions

**Figure 3:**
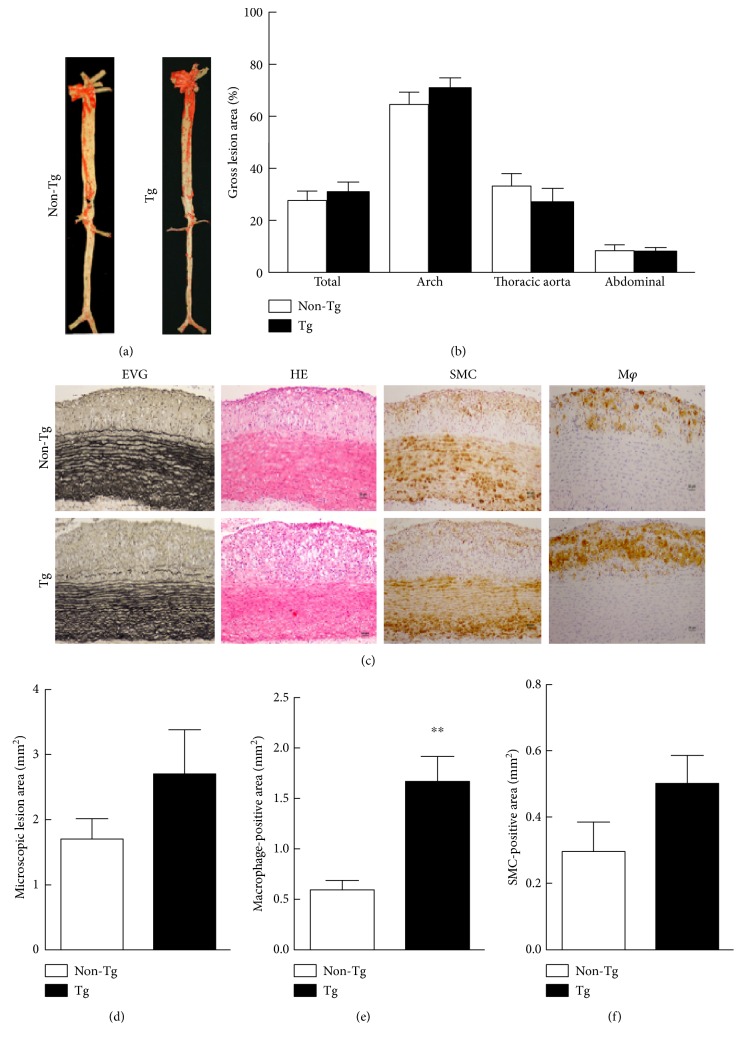
Representative aortic atherosclerosis lesions and their quantitative analysis. (a) “Aortic trees” were stained with Sudan IV. (b) Quantitative analysis of the atherosclerotic arterial lesions. (c) Aortic sections were stained with Elastica van Gieson (EVG) and hematoxylin and eosin (H&E) or immunohistochemically stained with Abs against macrophages (M*φ*) or smooth muscle cells (SMCs). The quantitative analysis of the aortic arch lesion area (d), the cellular composition of the M*φ*, and SMCs are shown at the bottom (e, f). *n* = 12 for each group. Data are expressed as the mean ± SEM. ^∗∗^*P* < 0.01 versus non-Tg.

Compared to the atherosclerotic lesions of the control group, increased expression of hCETP did not significantly affect the gross atherosclerotic lesions in Tg rabbits (Figure 3(a)). In addition, there was no significant difference in all parts of the rabbit aorta, including the aortic arch and the thoracic and abdominal aortas (Figure 3(b)). Representative micrographs of the aortic arch lesions of each group stained with EVG and H&E or immunohistochemically stained with Abs against SMC a-actin and RAM11are shown in Figure 3(c). Apparently, Tg rabbits showed increased tendency of intimal lesions along with enhanced SMCs and macrophage accumulation compared with that in non-Tg littermates (Figures 3(d) and 3(e)). However, only macrophage-positive areas were statistically significantly increased by 2.8-fold (*P* < 0.001) in Tg rabbits (Figure 3(e)).

## 4. Discussion

The potential atherogenicity of CETP relates to its ability to transfer cholesteryl esters from the antiatherogenic HDLs to the proatherogenic very low-density lipoproteins and LDLs [29–32]. However, there is also evidence that CETP may be involved in reverse cholesterol transport (transfer of cholesterol from peripheral cells through the plasma to the liver) [33]. Rare mutations leading to reduced function of CETP have been linked to accelerated atherosclerosis [34]. Genetic deficiency of CETP in rabbits has beneficial effects on enhancing HDL function and reducing atherosclerosis [35]. Thus, theoretically, CETP may be either proatherogenic or antiatherogenic. In this study, we successfully created Tg rabbits that expressed hCETP in the liver. Our present study showed that increased hepatic hCETP enhanced macrophage-derived foam cell accumulation in the lesions in Tg rabbits fed an HCD, even though there was no significant difference in the gross atherosclerotic lesions. Because the rabbits were fed with a cholesterol diet for 16 weeks, the main lesions are those of fatty streaks which are composed of macrophage-derived foam cells with a small number of SMCs. In human patients, those complicated lesions (such as plaque stenosis and rupture) leads to myocardial infarction. Therefore, it is necessary to investigate whether increased CETP can also affect plaque vulnerability in the future. For such a purpose, we need to feed the rabbits with a cholesterol diet for a longer time such as 28 weeks [28]. Atherosclerosis is a chronic disease process characterized by the focal subendothelial accumulation of apolipoprotein-B-containing lipoproteins, immune and vascular wall cells, and extracellular matrix [36]. The lipoproteins acquire features of damage-associated molecular patterns and trigger first an innate immune response, dominated by monocyte-macrophages, and then an adaptive immune response [37]. There are many studies showing that there are a number of autoantibodies existed in either plasma or atherosclerotic lesions that may initiate and participate in the development of atherosclerosis. High levels of antiphospholipid autoantibodies, antiphosphorylcholine autoantibodies, anti-LDL autoantibodies, and anticyclic citrullinated protein autoantibodies have been shown to be associated with increased cardiovascular risk [38]. Although our studies showed that increased CETP expression increased subendothelial accumulation of macrophages, pathophysiological significance of this finding in terms of macrophage infiltration and/or proliferation or autoantibody formation during the progression of atherosclerosis remains to be addressed in the future study. It was reported that dyslipidemic patients would have an elevated CETP concentration and/or an accelerated rate of net transfer of cholesteryl esters from HDL to apoB-containing lipoproteins as well as accelerated atherosclerosis [39]. CETP may be deleterious for atherosclerosis, but it is also likely that high levels of CETP are the result rather than the cause of dyslipidemia [40]. In our study, Tg rabbits with a higher CETP concentration had low levels of HDL-C and high levels of TGs but did not exhibit a significant effect on gross lesion area of aortic atherosclerosis. These observations may have implications for research into CETP inhibitors and the role of HDL-C in atherosclerosis.

Therapeutic intervention targeting HDL was once a major focus of research on the treatment of atherosclerotic disease [41]. The HDL-mediated removal of excess free cholesterol from macrophage foam cells is thought to play a major role in the protection against the development of atherosclerosis, which may have a possible beneficial effect on macrophage foam cell formation [42]. Large cholesteryl esters-rich HDL particles from 4 subjects with complete CETP deficiency showed an increased ability to promote cholesterol efflux from macrophage foam cells [43]. Inhibition of CETP by Torcetrapib increases macrophage cholesterol efflux to HDL [44]. In our study, high expression of the CETP gene was inborn, and the HCD was used as an arteriosclerotic auxiliary to further explore the roles of the CETP gene in the development of atherosclerosis and plaque formation. Immunohistochemical staining was performed to analyze the plaque components. We found that increased expression of the CETP promoted macrophage-derived foam cell formation in Tg rabbits. A high level of CETP mRNA and CETP concentration did not lead to a significant increase in the plasma LDL-C levels but did cause an obvious reduction in the HDL-C levels. In spite of this, there are several limitations in the current study. For example, it is not known whether CETP promotes accumulation of macrophage-derived foam cells in atherosclerotic lesions through inhibiting cholesterol efflux from macrophages, and other molecular mechanisms should be examined in the future. Furthermore, the number size of Tg and non-Tg rabbit size used in the current study was rather limited therefore whether these results can be directly translated into humans required more vigorous investigation. Finally, it remains to be established whether increased CETP expression can be used for treating atherosclerosis in humans. In conclusion, increased hepatic expression of hCETP in Tg rabbits increased macrophage-derived foam cell accumulation potentially via the reduction of HDL-C levels. The present studies may have implications for CETP inhibition in atherosclerosis.

## Figures and Tables

**Table 1:** The body weight, weight of the major organs, blood pressure, and heart rate in Tg and non-Tg littermates at the end of the experiments. Data are expressed as the mean ± SEM, *n* = 7 for each group.

	Non-Tg	Tg
Body weight (Kg)	3.40 ± 0.23	3.55 ± 0.21
Heart weight (g)	5.88 ± 1.05	6.44 ± 0.88
Kidney weight (g)	14.70 ± 1.94	15.78 ± 3.14
Liver weight (g)	96.92 ± 13.95	107.62 ± 9.07
SBP (mmHg)	99.5 ± 5.8	96.7 ± 7.8
DBP (mmHg)	87.6 ± 5.4	85.7 ± 6.6
Heart rate (BPM)	299.2 ± 13.4	248 ± 14.6

SBP: systolic blood pressure; DBP: diastolic blood pressure; BMP: beats per minute.

## Abbreviations

CETP: Cholesteryl ester transfer protein
EVG: Verhoeff van Gieson
HCD: High cholesterol diet
H&E: Hematoxylin and eosin
HDL-C: High-density lipoprotein cholesterol
LDL-C: Low-density lipoprotein cholesterol
M*φ*: Macrophages
RCT: Reverse cholesterol transport
SMC: Smooth muscle cell
TC: Total cholesterol
TG: Triglyceride.
