# Corrigendum to “Overexpression of Cholesteryl Ester Transfer Protein Increases Macrophage-Derived Foam Cell Accumulation in Atherosclerotic Lesions of Transgenic Rabbits”

**DOI:** 10.1155/2018/6904538

**Published:** 2018-04-15

**Authors:** Shoucui Gao, Xiaojing Wang, Daxin Cheng, Jiayan Li, Lu Li, Linwu Ran, Sihai Zhao, Jianglin Fan, Enqi Liu

**Affiliations:** ^1^Research Institute of Atherosclerotic Disease, Xi'an Jiaotong University Cardiovascular Research Center, Xi'an, Shaanxi 710061, China; ^2^Laboratory Animal Center, Xi'an Jiaotong University Health Science Center, Xi'an, Shaanxi 710061, China; ^3^Laboratory Animal Center, Ningxia Medical University, Ningxia 750004, China; ^4^Department of Molecular Pathology, Interdisciplinary Graduate School of Medicine and Engineering, University of Yamanashi, Yamanashi 409-3898, Japan

In the article titled “Overexpression of Cholesteryl Ester Transfer Protein Increases Macrophage-Derived Foam Cell Accumulation in Atherosclerotic Lesions of Transgenic Rabbits” [1], there were errors in one of the author names and in the Acknowledgments section.

The name of the third author was given incorrectly as Daxing Cheng. The author's name should have been written as Daxin Cheng. The revised author list is shown above.

In the Acknowledgments section, the text reading “This work was supported in part by the National Natural Science Foundation of China (no. 8107025 and 81370379)” should be corrected to “This work was supported in part by the National Natural Science Foundation of China (nos. 81070250 and 81370379).”
